# Effects of Host Plants on Bacterial Community Structure in Larvae Midgut of *Spodoptera frugiperda*

**DOI:** 10.3390/insects13040373

**Published:** 2022-04-11

**Authors:** Ya-Ping Chen, Ya-Hong Li, Zhong-Xiang Sun, E-Wei Du, Zhi-Hui Lu, Hao Li, Fu-Rong Gui

**Affiliations:** 1 State Key Laboratory for Conservation and Utilization of Bioresources in Yunnan, Yunnan Agricultural University, Kunming 650201, China; cyp83@ynau.edu.cn (Y.-P.C.); szx@ynau.edu.cn (Z.-X.S.); duewei123@sina.com (E.-W.D.); zhl223330311@163.com (Z.-H.L.); lh13695692952@126.com (H.L.); 2 Yunnan Plant Protection and Quarantine Station, Kunming 650034, China; liyahong20070820@163.com

**Keywords:** *Spodoptera frugiperda*, invasive insect, gut bacteria, host plant, 16S rRNA, toxic secondary metabolites

## Abstract

**Simple Summary:**

The gut microbiota plays an important role in insect physiology and behavior. The interaction among the different structures of gut bacterial community in the fall armyworm (FAW), *Spodoptera frugiperda*, and different host plants, and whether these different gut bacteria are responsible for the rapid spread of FAW to a variety of host plants after invasion are largely unexplored. In the present paper, we used a culture-independent approach targeting the bacterial 16S rRNA gene of gut bacteria of the 5th instar larvae of FAW fed on four different host plants. It aimed to analyze the effects of host plants on gut bacteria abundance, community structure and metabolic function. We found that host plants exerted considerable effects on the structure and composition of the gut bacteria in FAW and the differences among the four groups identified were significant. They were related to the detoxification and adaptation of FAW to toxic secondary metabolites of the host plant. These differences enabled the gut microbiome to perform different functions. This study lays a foundation for further studies on the function of intestinal bacteria in FAW and the adaptive mechanism to the host.

**Abstract:**

The fall armyworm (FAW), *Spodoptera frugiperda,* is one of the most important invasive species and causes great damage to various host crops in China. In this study, the diversity and function of gut bacteria in the 5th instar larvae of FAW fed on maize, wheat, potato and tobacco leaves were analyzed through 16S rRNA sequencing. A total of 1324.25 ± 199.73, 1313.5 ± 74.87, 1873.00 ± 190.66 and 1435.25 ± 139.87 operational taxonomic units (OTUs) from the gut of FAW fed on these four different host plants were detected, respectively. Firmicutes, Proteobacteria and Bacteroidetes were the most abundant bacterial phyla. Beta diversity analysis showed that the gut bacterial community structure of larvae fed on different host plants was significantly differentiated. At the genus level, the abundance of *Enterococcus* in larvae fed on wheat was significantly lower than those fed on the other three host plants. *Enterobacter* and *ZOR0006* were dominant in FAW fed on tobacco leaves, and in low abundance in larvae fed on wheat. Interestingly, when fed on Solanaceae (tobacco and potato) leaves which contained relative higher levels of toxic secondary metabolites than Gramineae (wheat and maize), the genera *Enterococcus, Enterobacter* and *Acinetobacter* were significantly enriched. The results indicated that gut bacteria were related to the detoxification and adaptation of toxic secondary metabolites of host plants in FAW. Further analysis showed that replication, repair and nucleotide metabolism functions were enriched in the gut bacteria of larvae fed on tobacco and potato. In conclusion, the gut bacterial diversity and community composition in FAW larvae fed on different host plants showed significant differences, and the insect is likely to regulate their gut bacteria for adaptation to different host plants.

## 1. Introduction

*Spodoptera frugiperda* (J. E. Smith) (Lepidoptera: Noctuidae) is a polyphagous insect pest native to the tropical and sub-tropical areas in America [1]. It is considered one of the most serious agricultural pests in the world, mainly due to the fact of its damage to various crops [2]. FAW larvae have a wide host range that involves more than 353 plant species belonging to 76 plant families [2], which includes maize, wheat, tobacco, rice, potato, and cotton [3,4,5]. The FAW is of two host-plant strains, the rice strain feeds preferentially on rice and several pasture grasses, and the corn strain primarily feeds on maize, cotton, and sorghum [6,7,8]. To unravel the possible mechanisms underlying host plant adaptations, extensive studies have revealed the comparison and difference between these two host-plant strains, including comparative analysis of whole genome sequencing [6], transcript expression plasticity [9], intraspecific differences in plant defense induction [10], and expression profiling of microRNAs [11]. The major types of control of *S. frugiperda* are currently biological and chemical, with chemical control still being dominant [12,13]. However, *S. frugiperda* has developed resistance to 41 commonly-used insecticides and may be responsible for its worldwide outbreaks [14]. This has further resulted in the increasing use of insecticides in the practical control of *S. frugiperda*, which is also very harmful to the environment.

The gut microbiota plays an important role in insect physiology and behavior, such as host plant selection [15], food digestion [16], host longevity and nutrition [17], immune response [18], insecticide resistance and environmental adaptability [19,20]. Symbiotic bacterial communities are involved in almost all insect life activities [21,22] and can facilitate the adaptation to new host plants, and to adapt to the environment [23,24].

Food is an important source of gut microorganisms [25,26], and host plants feeding could affect the species and functions of gut flora in herbivorous insects. The structure and diversity of gut microorganisms vary with host plants [27]. Plant secondary metabolites are one of the main barriers to insect feeding [28]. For instance, the concentrations of total phenols, tannins, flavonoids, and alkaloids in wheat leaves were found to be significantly lower than those in maize and potato leaves, and the concentration of nicotine in tobacco is higher than other plants [29]. Gut microorganisms which contain a variety of enzymes and can promote the synthesis of vitamins, as well as the absorption and utilization of fats and carbohydrates [30,31], are the driving force of the co-evolution between insects and their host plants [32]. However, the specific gut microorganisms in insects involved in adaptation to host plant secondary metabolites have not been identified extensively. 

At present, several gut bacterial strains associated with *S. frugiperda* have been isolated using culture-dependent methods, suggesting that the insect microbiota is influenced by environmental factors, such as the collecting site [33,34]. Jones et al. (2019) reported that the host plant was the major driver shaping gut microbiota in *S. frugiperda* and *Helicoverpa zea*. Two bacteria found in the oral secretions of *S. frugiperda* were shown to downregulate or upregulate the activities of plant defensive proteins in tomato and maize [35]. Gomes et al. found that *S. frugiperda* natural populations harbored a more diverse gut microbiota and a much higher diversity of bacteria capable of metabolizing insecticides than laboratory-selected populations [36]. The rapid invasion of *S. frugiperda* into various environments is closely related to its rapid adaptation to a variety of host plants, and its tolerance to toxic secondary metabolites. Lv et al. reported that the gut microbial community of *S. frugiperda* was affected not only by the host species, but also by different host treatments [37]. However, how *S. frugiperda* regulate gut flora to different host plants adaptation is not very clear. To date, the interaction between the different complexes of gut microflora in *S. frugiperda* and different host plants and whether this is responsible for the rapid spread of *S. frugiperda* to a variety of host plants are both largely unexplored. 

In this study, we used a culture-independent approach targeting the bacterial 16S rRNA gene of the gut bacteria in the 5th instar larvae of *S. frugiperda*, which fed on four different host plants containing different levels of toxic secondary metabolites (maize, wheat, potato, and tobacco). The aim was to analyze the effects of host plants on gut microfloral abundance, community structure, metabolic functions and the specific gut bacteria involved in host adaptation in *S.* *frugiperda*. These findings lay a foundation to understand the rapid adaptability of *S. frugiperda* to different host plants as influenced by the gut flora and also serve as a reference for the development of more effective and environmentally friendly *S. frugiperda* management strategies.

## 2. Materials and Methods

### 2.1. Insect Collection and Rearing Conditions

A laboratory population of *S. frugiperda* was established and maintained from insects that were originally collected from corn fields in Yuxi city, Yunnan Province, China (23°35′59.52″ N, 101°58′9.64″ E) in May 2019. *S. frugiperda* was reared indoors in artificial climate chambers (27 ± 0.5 °C, photoperiod 16 h L: 8 h D, RH 70% ± 5%) with artificial diets for 12 generations [38]. The seeds of four host plants (maize, wheat, tobacco and potato) were sown in flowerpots and grew in the greenhouse without the use of any pesticides. The larvae of *S. frugiperda* were fed for 5 generations with fresh maize leaves (SF-M), wheat leaves (SF-W), tobacco leaves (SF-T) and potato leaves (SF-P), respectively. Before feeding, the leaves surfaces were sterilized with 75% ethanol and rinsed in sterilized distilled water. 

### 2.2. Gut Dissection and 16S rRNA Gene Sequencing

The 5th instar larvae of *S. frugiperda* were obtained and placed on ice. After 30 min, they were washed with 75% alcohol for 120 s, rinsed with sterilized ultrapure water 3 times, and then dissected. The entire gut tissues of 3 larvae were collected in a centrifuge tube filled with 5 mL sterile PBS, fully shaken with an oscillator and mixed to prepare a suspension of intestinal contents. After quick freezing in liquid nitrogen, it was quickly stored in a −80 °C refrigerator for later use. Eight replicates were obtained. 

Total genomic DNA was extracted using a QIAamp DNA Stool Mini Kit (QIAGEN, CA, Hamburg, Germany) following the manufacturer’s instructions [39], and the DNA concentration was diluted to 1 ng/uL using sterile water. Bacterial 16S rRNA was specifically amplified with universal primers 27F (5′AGAGTTTGATCCTGGCTCAG-3′), 1492R(5′-CTACGGCTACCTTGTTACGA-3′). The PCR reaction conditions were pre-denaturation at 95 °C for 5 min, denaturation at 94 °C for 30 s, annealing at 52 °C for 30 s, extension at 72 °C for 1 min, 35 cycles and extension at 72 °C for 8 min. The size of PCR products was confirmed by agarose gel electrophoresis, and the amplified products were sent for sequencing. 16S rRNA gene sequencing targeting the v3–v4 region was performed on Illumina MiSeq/HiSeq to obtain raw data. This involved splicing into a sequence according to the overlapping relationship between the original sequences, performing quality control filtering on the sequence quality and splicing effect and splicing each sample sequence to obtain the splicing sequence, also known as the original tag data; high-quality tag data (clean tags) was obtained by filtering. Subsequently, by identifying and removing chimera sequences, effective data (effective tags) were finally obtained. Then the raw data underwent quality filtering to obtain valid data for each sample. Trimmomatic (version 0.35) was used to remove sequences less than 50 bp in length. Flash (version 1.2.11) was used to split joint and obtain complete paired-end sequences [15]. Split_libraries (version 1.8.0) was used to remove sequences less than 200 bp in length, to obtain clean tags. 

### 2.3. Operational Taxonomic Unit (OTU) Clustering and Annotation

All effective tags of samples were clustered (identity was 97% by default) to form OTU [40]. Representative OTU sequences were selected and compared to the ribosomal RNA database to obtain species annotation information [41]. Based on the annotation information of species, the chloroplasts, mitochondria and OTU that were not annotated to the boundary level and the tags contained therein were removed. Effective tags of different samples, tags with frequency of 1, tags with annotation, tags with annotation of chloroplast and mitochondria, and the number of effective OTU obtained by each sample were counted. Based on OTU abundance and annotation information, the relative abundance of microbial taxa at different taxonomic level (phylum, class, order, family and genus) was calculated for each sample. In addition, the effective sequencing data of 16S rDNA and the proportion of gut bacteria species of *S. frugiperda* which fed on the four kinds of host plants were counted.

### 2.4. Sequencing Data Analyses and Function Prediction

Alpha diversity reflects the abundance and species diversity of sample species, and commonly used measures are Chao1, Ace, Shannon, and Simpson [42]. The first two are often used to explain species abundance, while the latter two are used to measure species diversity. The *t*-test was used to compare the alpha diversity of the *S. frugiperda*, and significant difference was determined at *p* < 0.05. A Venn diagram was constructed to show common and unique OTUs of *S. frugiperda* fed on the four host plants.

Beta diversity analyses aims to compare the differences in the similarity of species diversity of individual samples, that is, the differences in bacterial composition among samples [42]. The similarities and differences among different groups were analyzed using the non-metric multidimensional scaling (NMDS) method. Anosim similarity analysis was used to judge whether the grouping was meaningful. 

Linear discriminant effect size (LEfSe) is an analysis method based on Linear discriminant analysis (LDA), usually chosen to set LDA score >2 as the biomarker screening standard, to obtain significant species and biomarkers with differences between groups [42]. LEfSeand similarity percentage (SIMPER) were used to determine species with significant differences in abundance between groups (i.e., biomarker). 

The functional prediction analysis of 16S rRNA sequencing data was carried out using PICRUSt. STAMP was used to visualize the results, including Kyoto Encyclopedia of Genes and Genomes (KEGG). One-way analysis of variance (ANOVA) was used to analyze the comparison between two groups (*p* < 0.05 indicates significant difference). The NTsys software (version 2.10) was used to perform UPGMA (unweighted pin-group method with arithmetic mean) clustering analysis for the samples, based on average taxonomic distance.

### 2.5. Statistical Analyses

The statistical software, R (version 4.1.3), was used for analyses. The OTU data between groups are expressed as the mean ± standard error, ANOVA was used for comparison between groups and linear discriminant analysis effect size (LEfSe) was used for analysis of differences in community structure. Statistically significant difference was determined at *p* < 0.05.

## 3. Results

### 3.1. Sequencing Data Statistics and Clustering

**Figure 1:**
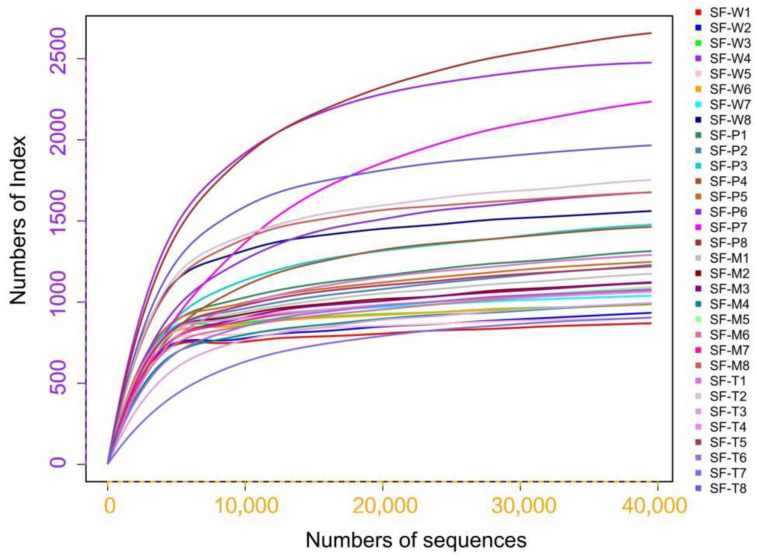
Rarefaction curve of intestinal bacterial species in the 5th instar larvae of *S. frugiperda.* Eight replicates were set up for each group, with the numerals 1–8 after the sample name representing different replicates.

The clean reads (SF-W: 70,602.38 ± 3294.11; SF-M: 73,055.25 ± 515.94; SF-P: 73,560.88 ± 571.33; SF-T: 73,838.75 ± 581.67) were retained after quality control and all reads were longer than 200 (Table S1). All effective tags were clustered into OTUs (SF-W: 1324.25 ± 199.73; SF-M: 1313.50 ± 74.87; SF-M: 1873.00 ± 190.66; SF-T: 1435.25 ± 139.87) (Table 1). For each of the four treatment groups, the sequencing depth was sufficient to reveal most of the bacterial diversity in larval guts (Figure 1).

**Figure 2:**
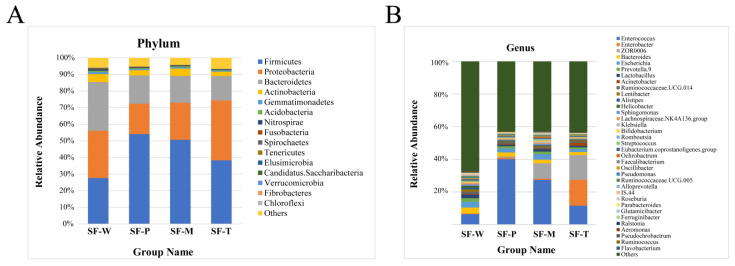
Relative abundance of bacterial taxa in the intestine of larval *S. frugiperda* fed on four host plants at different classification levels. (**A**): phylum level; (**B**): genus level.

Based on OTU abundance and annotation information, they were annotated into 45 phyla and 690 genera. At the phylum level, the most abundant bacterial phyla identified across the *S. frugiperda* guts were Firmicutes, Proteobacteria, and Bacteroidetes regardless of host plants, including other phyla, such as Actinobacteria, Gemmatimonadetes, Acidobacteria and Nitrospirae (Figure 2A), which are considered constituents of the core gut microbiota of *S. frugiperda*. The abundance of Firmicutes was the highest among the gut flora of larvae fed on potato leaves and maize leaves (54.08% in SF-P and 50.65% in SF-M). Phyla of the SP-W were evenly distributed, with Firmicutes accounting for 27.68%, Proteobacteria for 28.47%, and Bacteroidetes for 29.26% (Figure 2A). 

At the genus-level, the most abundant bacterial taxa were *Enterococcus*, *Enterobacter*, *ZOR0006*, *Bacteroides*, and *Escherichia* (Figure 2B). Phylogenetic analysis revealed the diversity of the species composition of the community and its phylogenetic relationship (Figure S1). Although the composition of gut microbiota in these four populations was similar, the abundance was different. The abundance of *Enterococcus* in SF-P (40.12%) was much higher than that in the other three populations, while *Enterococcus* was the lowest within SF-W (6.38%) (Figure 2B); *Enterobacter* was the most abundant genus in SF-T (15.84%), which represented 0.90% in SF-P, 0.75% in SF-M and 0.46% in SF-W, respectively (Figure 2B). 

### 3.2. Alpha Diversity of Gut Flora

Figure 1 shows that as the number of samples increased the curve tended to be flat, indicating that the amount of sequencing data was reasonable. Alpha diversity is the analysis of species richness and diversity in a sample. The larval gut bacteria of all samples had high richness and diversity (Table 2). Shannon and Simpson diversity metrics demonstrated that SF-W had the highest bacterial diversity, with good uniformity (Table 2). 

**Figure 3:**
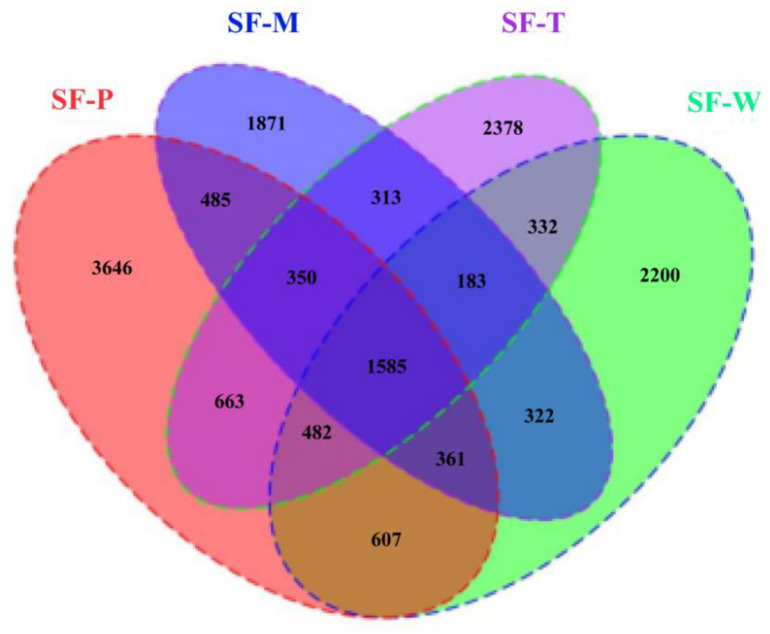
Venn diagram of taxonomical operational units (OTUs) of the gut bacterial of larval *S. frugiperda*.

Statistical analysis unveiled the common and unique OUTs between *S. frugiperda* fed on the four host plants (Figure 3). Overall, only 1585 of the total OTUs identified in different samples were present in all the individuals (representing 0.1%). There were some OTUs that did not belong to any of the four treatment groups (2200, 3464, 1871 and 2378 in SF-W, SF-P, SF-M and SF-T, respectively). 

### 3.3. Beta Diversity of Gut Flora

**Figure 4:**
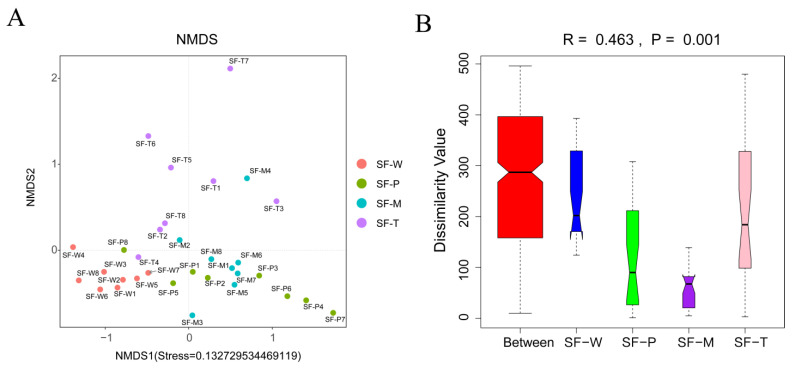
Beta diversity analysis of gut flora. (**A**) NMDS analysis: The points in the figure represent the samples, and the distance between the points indicates the degree of difference; the stress < 0.2 indicates that NMDS analysis had certain reliability. (**B**) Anosim analysis: The R value was (−1,1). R > 0 indicates that the difference among groups is greater than that within groups, and R < 0 indicates that the difference within groups is greater than that among groups. The reliability of statistical analysis is expressed by *p*-value, and *p* < 0.05 indicates statistical significance.

NMDS based on Bray–Curtis dissimilarities revealed that the composition of gut flora OTUs in *S. frugiperda* fed on different host plants overlapped, although some significant dissimilarities were recorded at the genus level (stress = 0.13273) (Figure 4A). The eight replications of SF-W and SF-T clustered together respectively and distributed in different regions, while the gut flora of SF-M and SF-P was relatively concentrated but showed differences. These results indicated the strong effect of host plants on the gut microbiota of *S. frugiperda* larval. To further determine whether the grouping was meaningful, we used Anosim similarity to test whether the differences among groups (two or more groups) were significantly greater than that within groups. The results confirmed that the grouping was reasonable (R = 0.463, *p* = 0.001) (Figure 4B). There were significant differences among the 4 groups, and the inter-group differences were much greater than the intra-group differences.

### 3.4. Differences in Community Structure of Gut Flora

**Figure 5:**
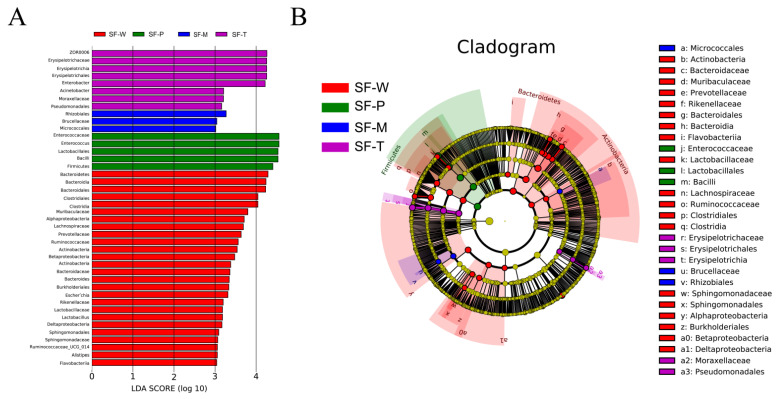
Microbial taxa associated with different host plants. (**A**) Association of specific microbiota taxa with the group of 4 host plants by linear discriminant analysis (LDA) effect size (LEfSe). (**B**) The taxonomic cladograms obtained from LEfSe analysis of 16S rDNA sequences are shown. Small circles highlighted in different colors (red, green–blue or purple) in the diagram represent the taxa that were significantly elevated in the respective group. Yellow circles indicate taxa that were not significantly differentially represented (*p* > 0.05).

To identify which species exhibited significant differences in abundance between groups (i.e., biomarker), we performed the LEfSe analysis based on nonparametric rank-sum test. The results revealed that the number of taxa (LDA > 3) was 25, 3, 5 and 8 among the SF-W, SF-M, SF-P, and SF-T groups, respectively (Figure 5A). There were more biomarker species in SF-W group, but fewer biomarker species in SF-M group. Due to the subordination between biomarkers at different classification levels, the results of the LEfSe analysis were sorted out in Table S2. Based on the results obtained, an evolutionary branching tree was drawn to show the microbial community or species structure with group differences from phylum to genus. Thirty genera showed significant differences among the four groups, with 19 of them more abundant in *S. frugiperda* fed on wheat leaves, 5 that fed on tobacco leaves, and 3 that fed on maize leaves and potato leaves (Figure 5B).

### 3.5. Functional Prediction of Gut Flora and Their Difference 

**Figure 6:**
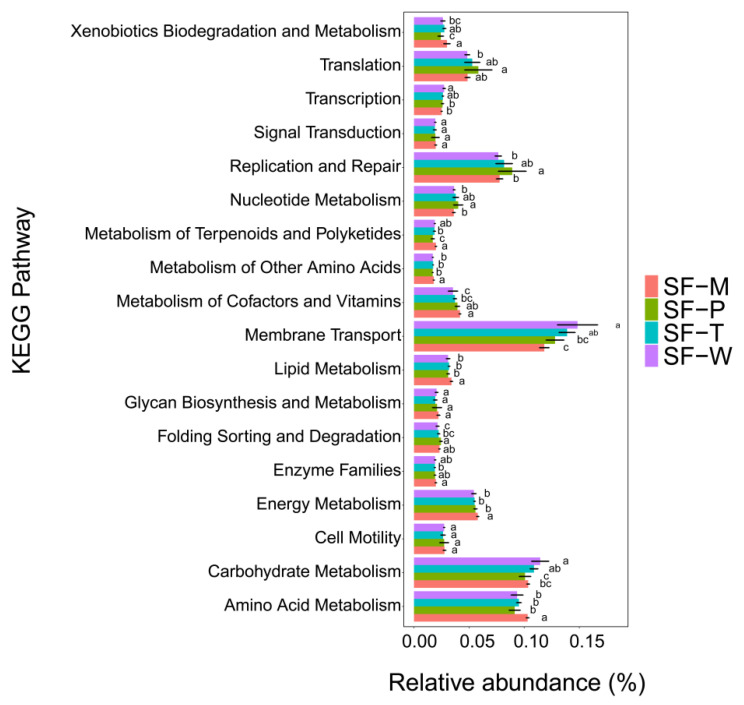
KEGG function prediction and function difference of the gut flora between feeding on four host plants. Different lowercase letters above bars indicate significant differences among samples of the same gene group (*p* < 0.05, One-way ANOVA).

The functions and pathways of gut bacteria in *S. frugiperda* were predicted. Among them, xenobiotics biodegradation and metabolism, metabolism of terpenoids and polyketides, metabolism of other amino acids, metabolism of cofactors and vitamins, lipid metabolism, enzyme families, energy metabolism and amino acid metabolism were higher in *S. frugiperda* fed on maize leaves compared to those in the other groups (Figure 6). The gut microbial genes of *S. frugiperda* fed on maize leaves mainly performed nutritional metabolic functions. Replication and repair, and nucleotide metabolic functions were enriched in gut flora of larvae fed on tobacco and potato, respectively (Figure 6). Since potato has a higher flavonoid content [16], the *S. frugiperda* fed on it had the lowest abundance of genes that participated in the metabolism of terpenoids and polyket (Figure 6).

## 4. Discussion

Many important cultivated crops are host plants for *S. frugiperda*. Yunnan was the first place where *S. frugiperda* invaded in China. Its adaptation to important crops in Yunnan may affect its spread. This study investigated the mechanism underlying the adaptation of *S. frugiperda* to different host plants. Host plants play a critical role in shaping the structure and function of the insect gut microbiome [43]. In this study, we used a culture-independent approach targeting the bacterial 16S rRNA gene of gut microflora in the 5th instar larvae of *S. frugiperda* fed on four different host plants, which included two Poaceae plants (maize and wheat) and two Solanaceae plants (potato and tobacco), under laboratory conditions and without any pesticide exposure. Our findings revealed that the differences in microbial communities were significantly influenced by different host plants. Similar results were also confirmed in a previous study for some other host plants (corn, wild oat, oilseed rape, pepper and artificial diet) [37]. Our study focused more on the possible correlation of these differences with plant species or their toxic secondary metabolites.

Our findings revealed that gut bacterial richness was significantly higher in *S. frugiperda* fed on wheat leaves (Table 2). Wheat is the most suitable host plant of *S. frugiperda* in laboratory conditions. However, maize is the most attacked crop in the world by this pest [2]. This is mainly due to the fact that *S. frugiperda* is native to tropical America, while wheat is mostly grown in cooler regions. With the development of new agricultural facilities, many crops are planted throughout the year. *S. frugiperda* may potentially have more host plants and therefore can cause greater damage.

A previous study on *Spodoptera litura* suggested that omnivorous insects need more carbohydrate hydrolases to assist the digestion and degradation of food, and the enzymes that degrade cellulose, hemicellulose, and pectin are mainly encoded by the bacterial genes in Firmicutes and Proteobacteria [44]. Our study showed that the dominant bacteria found in the *S. frugiperda* larval midgut belonged to these phyla (Figure 1), which is consistent with similar results obtained from studies on other Lepidoptera [45,46,47]. Proteobacteria have been reported to degrade insecticides and had the highest abundance in the gut flora of field-collected larvae [48]. In our study Firmicutes was identified as the most abundant bacterial phylum, a result that is similar to that of Lv et al. [37]. Also, the host plants of *S. frugiperda* in our study were grown without using any pesticides. At the genus level, the most abundant bacterial taxa were *Enterococcus*, *Enterobacter, ZOR0006*, *Bacteroides,* and *Escherichia* (Figure 1). *Enterococcus* and *Enterobacter* are mainly implicated in the degradation of the plant cell wall, which can degrade the cell wall into available nutrients [49]. In addition, *Enterococcus* plays an important role in metabolizing plant toxic secondary metabolites. The intestinal microbiomes of *Brithys Crini* and *Hyles Euphbiae,* two lepidopteran species feeding on toxic plants rich in alkaloid were studied. Although they belong to different lepidoptera families, they were shown to have similar bacterial communities, surprisingly dominated by *Enterococcus* [27]. The abundance of *Enterococcus* was notably lower in larvae that fed on wheat. The reason could be that compared to the other three plant species, the leaves of wheat had fewer toxic substances for secondary defense [29]. It may be the same reason why SF-W had the highest bacterial diversity in Shannon and Simpson diversity metrics (Table 2) since the secondary metabolites had little inhibitory effect on the gut flora. Although SF-P had more species (Table 1), its dominant species were more concentrated and less uniform than that of SF-W and SF-M (Figure 4). Potato and tobacco are Solanaceae plants, whose levels of secondary metabolites such as total phenols, tannins, flavonoids, and alkaloids and nicotine are higher than other plants [29], which may exert selective pressure on the gut bacteria of herbivore larvae, to influence the abundance of some species.

LEfSe analysis can search for species with statistical differences and help find species with significant differences in abundance between groups (i.e., Biomaker). The biomarker may indicate the role of gut flora in the adaptation of *S. frugiperda* to different host plants. There was a significantly high abundance of *Enterobacter* among the gut flora of SF-T (Figure 2B). *Enterobacter* can synthesize amino acids and degrade plant toxic secondary metabolites in *Plutella xylostella*. *Enterobacter* are able to effectively degrade toxic phenols that enter the midgut during the digestion of food by *Plutella xylostella* (Linnaeus) [50], and the high abundance of *Enterobacter* accelerates the development of resistance to insecticides [20]. The ability of gut microbes to metabolize defensive secondary plant substances and associated chemicals in insecticides, and to potentially inducing resistance in their insect hosts, is partly responsible for the development of insecticide resistance and the increasing suitability to a variety of host plants of many insect species. Furthermore, many species of *Acinetobacter* can degrade nicotine [20], thus directly reducing the toxicity of nicotine to insects and enhancing the tolerance of *S. frugiperda* to tobacco [51]. In the SF-P, *Enterococcus* was one of the species with significant differences in abundances (Figure 2B). It may have had a positive effect on the resistance of *S. frugiperda* to solanine in potato. For instance, *Enterococcus* been isolated from *Manduca sexta* (Lepidoptera, Sphingidae), a specialist species that feeds on toxic Solanaceae, rich in phenolic derivatives of caffeic acid [44]. *Enterococcus* in the gut of gypsy moth larvae was reported to reduce the insecticidal activity of BT [52], while that in the gut of *Manduca sexta* and *P. xylostella* can reduce the insecticidal activity of Cry1Ac [53]. Some Burkholderia bacteria settle in the gut of *Riptortus pedestris* and assist the host to increase its resistance to the toxicity of pyridoxine, thereby facilitating the host survival under pesticide stress [54]. It was demonstrated that *S. frugiperda* was likely to show new host adaptability under the selection pressure of insecticides, which may enhance its adaptability to plants that are indigestible or contain lethal compounds [55]. Therefore, insecticide resistance–host adaptability should be studied in-depth to unravel the synergistic effects of gut microbes. And further studies are warranted to determine the relationship between specific chemicals in host plants and the gut microbiota of *S. frugiperda* and also to explore how resulting changes in the composition and abundance of gut microbiota affect insect behaviors.

PICRUST results showed that the wheat group was enriched in carbohydrate metabolism (Figure 6). The soluble sugars content in wheat leaves was higher than those in other plant leaves, so the *S. frugiperda* fed on wheat may have required a higher proportion of gut bacteria to assist in the synthesis of the carbohydrate [29]. Also, the relative abundance of terpenoids and polyketides metabolic genes in the maize and wheat treatment group were higher than that in the tobacco and potato treatment group, which might be related to the presence of ketone insecticides in maize leaves such as benzoxazosin [55].

This study lays a foundation for further studies on the function of gut bacteria in *S. frugiperda* and the adaptive mechanism to host plants. Gut microflora can contribute to the adaptability to different host plants and metabolic functions. The use of metagenome and metabonomics to study the dynamic response and changes in the gut microflora of *S. frugiperda* fed on different host plants may clarify the function of these microfloras in the relationship between *S. frugiperda* and its host plants. Therefore, a specific host plant can be targeted to reduce resistance to insecticides in insects by adjusting their intestinal flora structure, thus becoming a new, environmentally friendly pest control strategy.

## 5. Conclusions

To our knowledge, our study describes, for the first time, how host plants affect the gut bacteria of *S. frugiperda*. The results showed that the gut bacterial diversity and community composition in *S. frugiperda* larvae fed on different host plants showed significant differences which were related to the detoxification and adaptation of *S. frugiperda* to toxic secondary metabolites of the host plant. *S. frugiperda* is likely to regulate their gut flora to adapt to different host plants. To further clarify the function of gut bacteria of *S. frugiperda* and its relationship with host plants, we applied metagenomics and metabonomics to study the dynamic response and change process of the gut bacteria in *S. frugiperda* fed on different host plants. This was to determine the role played by the gut bacteria in the expansion of the host spectrum of *S. frugiperda.* This study lays a foundation for further studies on the function of gut bacteria in *S. frugiperda* and the adaptive mechanism to its host plants.

## Figures and Tables

**Table 1:** Statistics of effective sequencing data of bacterial 16S rRNA in the larval *S. frugiperda*.

Sample	Raw_Reads_R	Clean_Reads	Total_Tag	Taxon_Tag	Unique_Tag	OTU_Num.
SF-W	79,873.25 ± 546.44	70,602.38 ± 3294.11	63,671.63 ± 3377.64	62,599.25 ± 3342.82	971.13 ± 84.94	1324.25 ± 199.73
SF-M	80,458.00 ± 398.37	73,055.25 ± 515.94	65,966.75 ± 988.87	65,031.88 ± 1056.30	830.38 ± 71.51	1313.50 ± 74.87
SF-P	80,129.88 ± 521.60	73,838.75 ± 581.67	63,773.63 ± 876.75	62,810.50 ± 936.74	906.50 ± 82.30	1873.00 ± 190.66
SF-T	79,677.75 ± 476.71	73,560.88 ± 571.33	66,188.75 ± 1040.84	65,275.75 ± 1108.93	873.63 ± 106.75	1435.25 ± 139.87

The means of the estimated diversity indices are reported for each analyzed group of *S. frugiperda.*

**Table 2:** Alpha diversity indices of the bacteria in the gut of larval *S. frugiperda*.

	Shannon Index	Simpson Index	Chao1 Index	Ace Index
SF-W	8.90 ± 0.14 a	0.99 ± 0.00 a	1348.62 ± 184.46 a	1371.17 ± 177.76 a
SF-P	6.54 ± 0.59 b	0.81 ± 0.06 b	1906.92 ± 184.31 a	1862.35 ± 181.10 a
SF-M	6.87 ± 0.24 b	0.90 ± 0.02 ab	1383.32 ± 71.24 a	1344.99 ± 62.78 a
SF-T	6.31 ± 0.65 b	0.87 ± 0.04 ab	1471.53 ± 147.96 a	1390.33 ± 136.05 a
F	6.61	4.38	2.81	2.82
df	3	3	3	3
*p*	0.002	0.012	0.058	0.057

Different lowercase letters denote significant differences between groups (*t*-test, *p* < 0.05).

## Data Availability

The raw data supporting the conclusions of this article will be made available by the authors, without undue reservation, to any qualified researcher.

## Supplementary Materials

The following are available online at https://www.mdpi.com/article/10.3390/insects13040373/s1, Table S1: Statistics of the OTU numbers of bacteria in the gut of the 5th instar larvae of *S. frugiperda**;* Table S2: Biomarkers of bacteria in the gut of the 5th instar larvae of *S. frugiperda*; Figure S1: Neighbor-joining phylogenetic tree of the larval gut microbiota of *S. frugiperda*.
