# Supplementary material for: Effects of Host Plants on Bacterial Community Structure in Larvae Midgut of Spodoptera frugiperda
Source: Insects. 2022 Apr 11;13(4):373. doi: 10.3390/insects13040373 (PMC9031720; doi:10.3390/insects13040373)
Supplement: Supplementary file 1 [file insects-13-00373-s001.zip › insects-1653134-supplementary.pdf]

Table S1 Statistics of the OTU numbers of bacteria in the gut of the 5th instar larvae of *S. frugiperda*

sample	raw_reads_R	clean_reads	Total_tag	Taxon_Tag	Unique_Tag	OTU_num
SF-W1	79047	73373	66857	65567	1225	953
SF-W2	81579	75628	68142	67213	886	1048
SF-W3	79432	73055	65984	64682	1088	1092
SF-W4	78928	47864	40351	39492	843	2597
SF-W5	79578	73937	68059	67119	791	990
SF-W6	81913	76374	69638	68209	1219	1086
SF-W7	77380	71420	64855	64213	556	1130
SF-W8	81129	73168	65487	64299	1161	1698
SF-M1	78160	72903	67453	66556	805	1334
SF-M2	79710	71998	63550	62231	1142	1277
SF-M3	81069	73379	65547	64445	1082	1260
SF-M4	81586	74696	68952	68231	684	1125
SF-M5	80315	70744	68832	68129	674	1259
SF-M6	80512	72968	66759	65911	761	1207
SF-M7	81605	75338	66039	65314	573	1230
SF-M8	80707	72416	60602	59438	922	1816
SF-P1	81518	75452	64643	63617	980	1544
SF-P2	78307	71681	64100	63252	716	1429
SF-P3	81312	74694	65633	64648	907	1687
SF-P4	80861	74365	67497	66823	652	1661
SF-P5	81132	74837	60219	59218	961	1438
SF-P6	81068	74770	63372	62477	819	1846
SF-P7	78824	74035	64373	63545	808	2437
SF-P8	78017	70876	60352	58904	1409	2942
SF-T1	79657	74539	66430	65653	774	1453
SF-T2	78693	72322	62616	61491	1104	1951
SF-T3	81229	73602	68721	68003	637	1141
SF-T4	78224	73758	65576	64242	1334	1194
SF-T5	81893	76197	67855	66827	922	1377
SF-T6	79791	74222	69002	68168	789	1237
SF-T7	79776	73148	68237	67873	363	1013
SF-T8	78159	70699	61073	59949	1066	2116

The numbers 1-8 following the sample names represent different replicates, respectively.

Table S2 Biomarkers of bacteria in the gut of the 5th instar larvae of *S. frugiperda*

Group	Phylum	Class	Order	Family	Genus
SF-T	Finmicutes				ZOR0006*
	Firmicutes	Mollicutes	Mycoplasmatales	Erysipelotrichaceae*	
	Firmicutes	Erysipelotrichia*			
	Firmicutes	Erysipelotrichia	Erysipelotrichales*		
	Proteobacteria	Gammaproteobacteria	Enterobacterales	Enterobacteraceae	Enterobacter*
	Proteobacteria	Proteobacteria	Pseudomonadales	Moraxellaceae	Acinetobacter*
	Proteobacteria	Proteobacteria	Pseudomonadales	Moraxellaceae*	
	Proteobacteria	Proteobacteria	Pseudomonadales*		
ST-M	Proteobacteria	Alphaproteobacteria	Rhizobiales*		
	Proteobacteria	Alphaproteobacteria	Rhizobiales	Brucellaceae*	
	Actinobacteria	Actinobacteria	Micrococcales*		
SF-P	Finmicutes	Bacilli	Lactobacillales	Enterococcaceae*	
	Finmicutes	Bacilli	Lactobacillales	Enterococcaceae	Enterococcus*
	Finmicutes	Bacilli	Lactobacillales*		
	Finmicutes	Bacilli*			
	Firmicutes*				
SF-W	Bacteroidetes*				
	Bacteroidetes	Bacteroidia*			
	Bacteroidetes	Bacteroidia	Bacteroidales*		
	Firmicutes	Clostridia	Clostridiales*		
	Firmicutes	Clostridia*			
				Muribaculaceae*	
	Proteobacteria	Alphaproteobacteria*			
	Firmicutes	Clostridia	Clostridiales	Lachnospiraceae*	
	Bacteroidetes	Bacteroidia	Bacteroidales	Prevotellaceae*	
	Firmicutes	Clostridia	Clostridiales	Ruminococcaceae*	
	Actinobacteria	Actinobacteria*			
	Proteobacteria	Betaproteobacteria*			
	Bacteroidetes	Bacteroidia	Bacteroidales	Bacteroidaceae*	
	Bacteroidetes	Bacteroidia	Bacteroidales	Bacteroidaceae	Bacteroides*
	Proteobacteria	Betaproteobacteria	Burkholderiales*		
	Proteobacteria	Gammaproteobacteria	Enterobacterales	Enterobacteraceae	Escher'chia*
				Rikenellaceae*	
	Finmicutes	Bacilli	Lactobacillales	Lactobacillaceae*	
	Finmicutes	Bacilli	Lactobacillales	Lactobacillaceae	Lactobacillus*
	Proteobacteria	Deltaproteobacteria*			
	Bacteroidetes	Sphingomonadia	Sphingomonadales*		
	Bacteroidetes	Sphingomonadia	Sphingomonadales	Sphingomonadaceae*	
	Firmicutes	Clostridia	Clostridiales	Lachnospiraceae	Ruminococcaceae-UCG-014*
	Firmicutes	Clostridia	Clostridiales	Rikenellaceae	Alistipes*

Bacteroidetes	Flavobacteriia*

“*” represents the dominant biomarker of bacteria in the gut of the 5th instar larvae of *S. frugiperda*

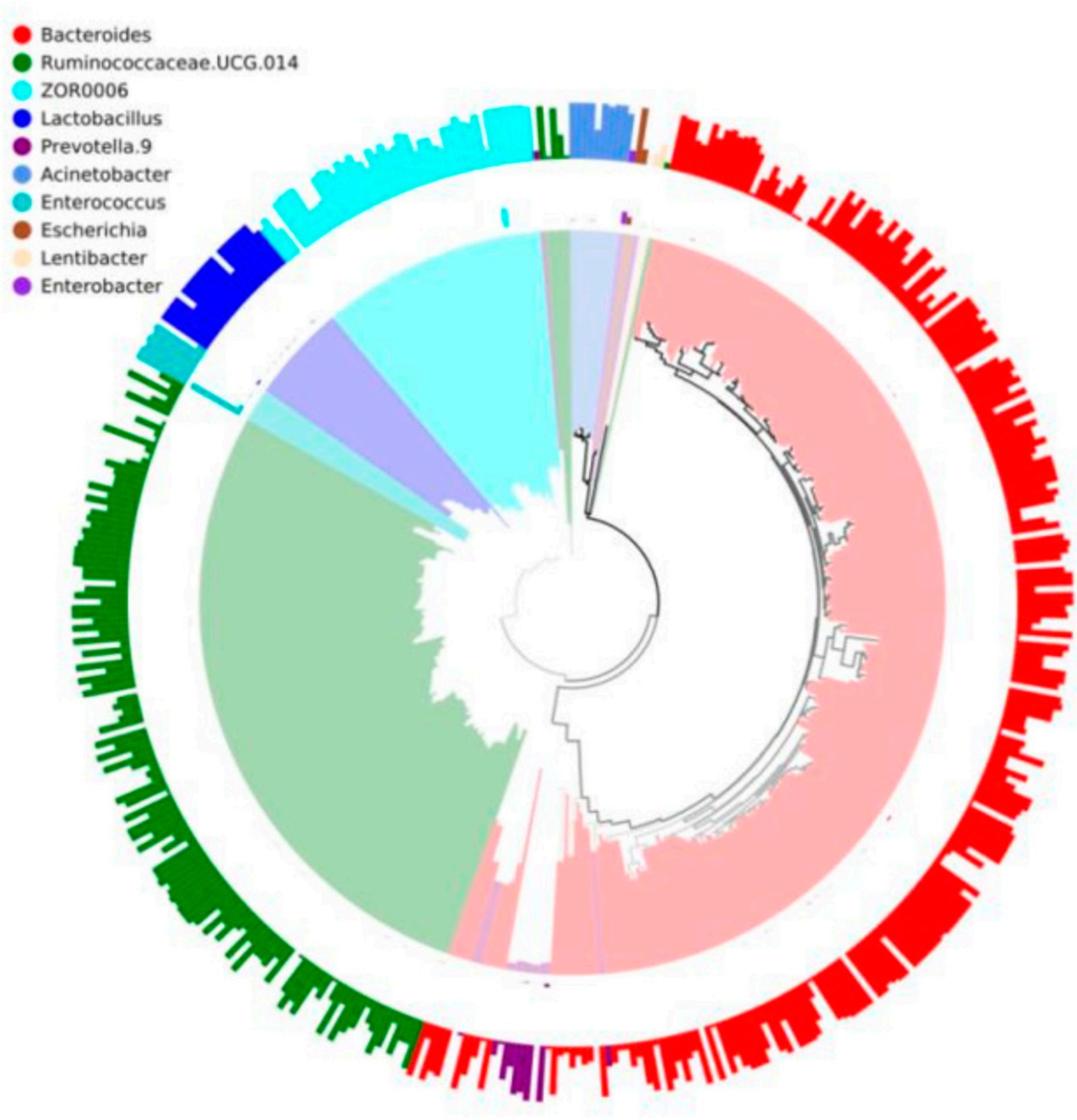

Figure S1. Neighbor-joining phylogenetic tree of the larval gut microbiota of *S. frugiperda*.
